# Supplementary material for: pH-Sensing G Protein-Coupled Receptor OGR1 (GPR68) Expression and Activation Increases in Intestinal Inflammation and Fibrosis
Source: Int J Mol Sci. 2022 Jan 26;23(3):1419. doi: 10.3390/ijms23031419 (PMC8835966; doi:10.3390/ijms23031419)
Supplement: Supplementary file 1 [file ijms-23-01419-s001.zip › ijms-1530325-supplementary.pdf]

Supplementary Material

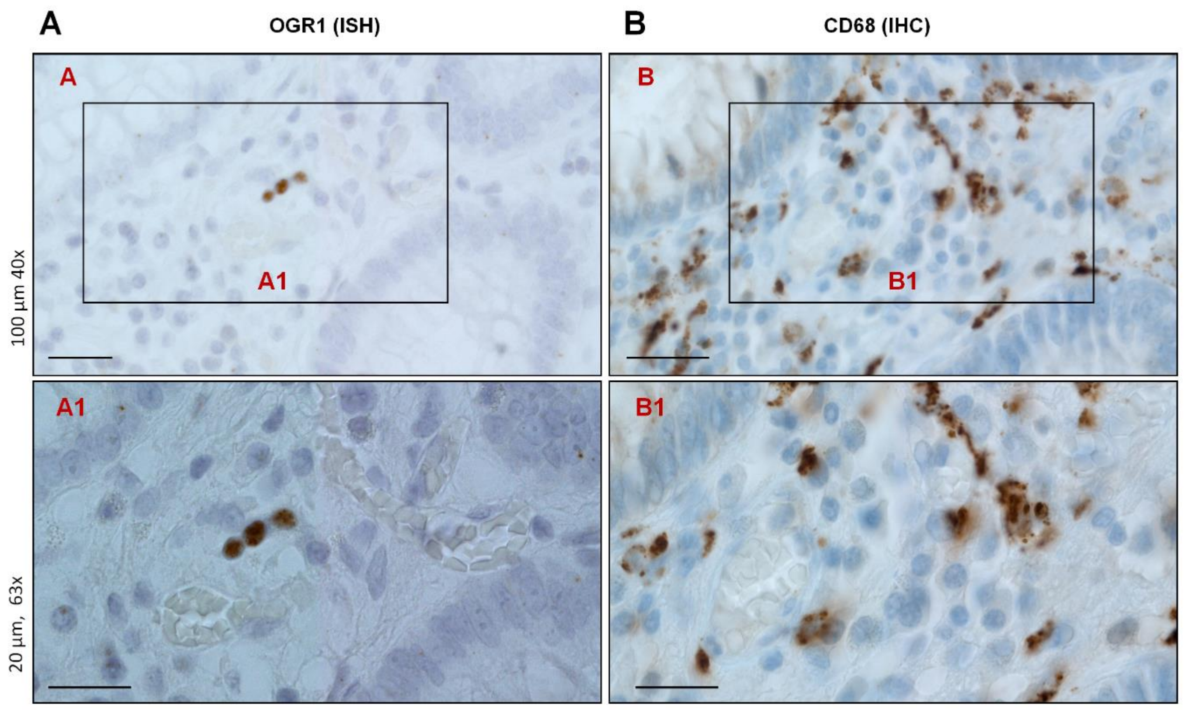

Suppl. Fig. 1. *In situ* hybridization (ISH) detection of OGR1 and immunohistochemical (IHC) detection of CD68 in colonic tissue. **A.** ISH staining of OGR1 mRNA (brown dots). **B.** IHC staining (brown colour) with anti-CD68 antibody, counterstaining with haematoxylin. Intestinal samples were taken from 5 non-IBD subjects and paired (non-inflamed and inflamed) samples from 5 CD patients and 5 UC patients. Representative images are shown from a CD patient, non-inflamed tissue. Scale bars: **A–B**. overview images 100 µm; **A1–B1**. detail images, 20 µm. CD: Crohn's disease.

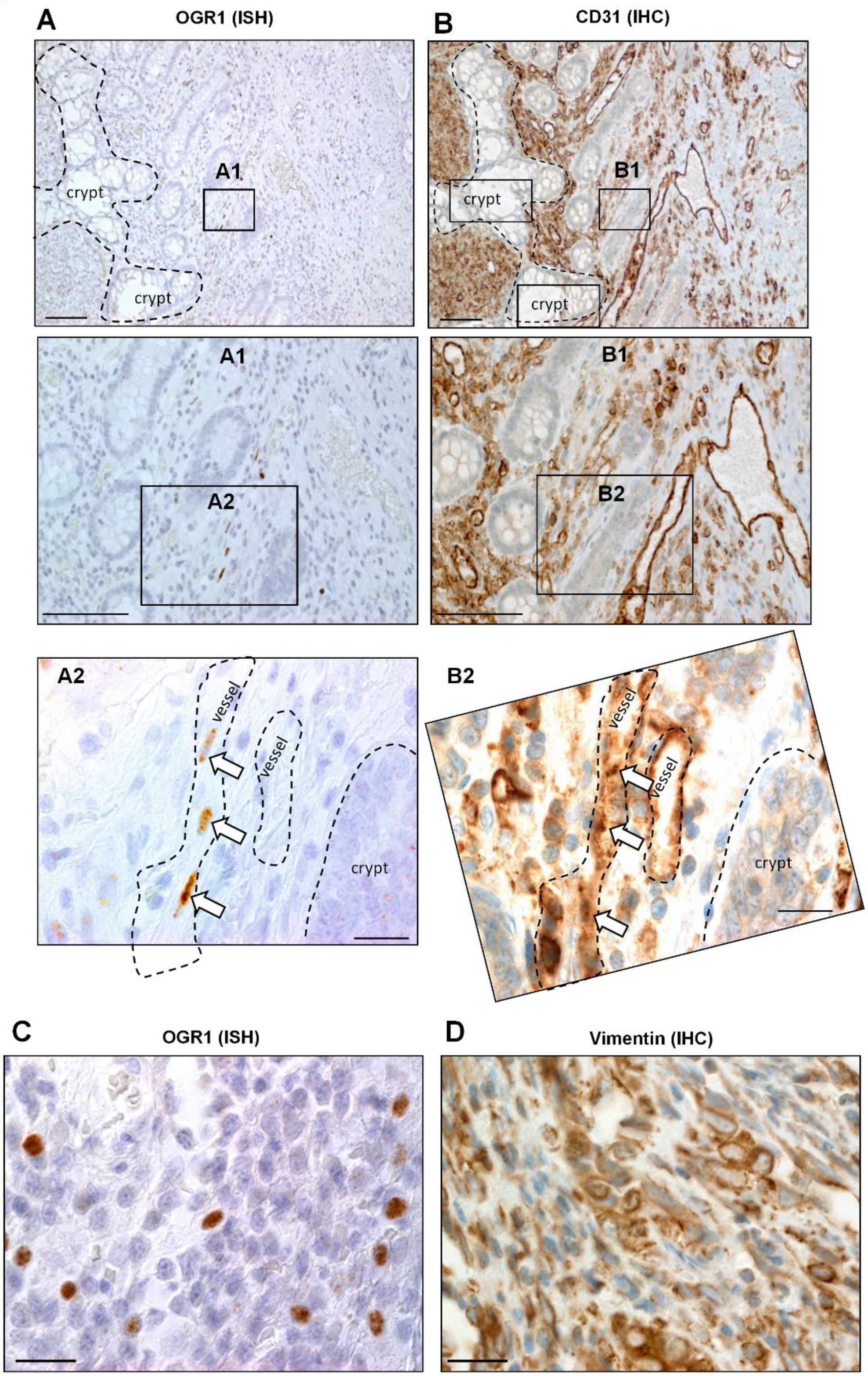

Suppl. Fig. 2. In situ hybridization (ISH) detection of OGR1 and immunohistochemical detection of CD31 and vimentin in colonic tissue. **A.** and **C.** ISH staining of OGR1 mRNA (brown dots). **B.** IHC staining (brown colour) with anti-CD31 antibody, counterstaining with haematoxylin. Scale bars: **A–B.** overview images 500 µm. **A1–B1.** images, 100 µm. **A2–**

B2. detail images, 20 µm. **D.** IHC staining (brown colour) of anti-vimentin antibody, counterstaining with haematoxylin. Scale bars: **C–D.** images, 20 µm. Intestinal samples were taken from 5 non-IBD subjects and paired (non-inflamed and inflamed) samples from 5 CD patients and 5 UC patients. Representative images are shown from a CD patient, non-inflamed tissue. IBD: inflammatory bowel disease.

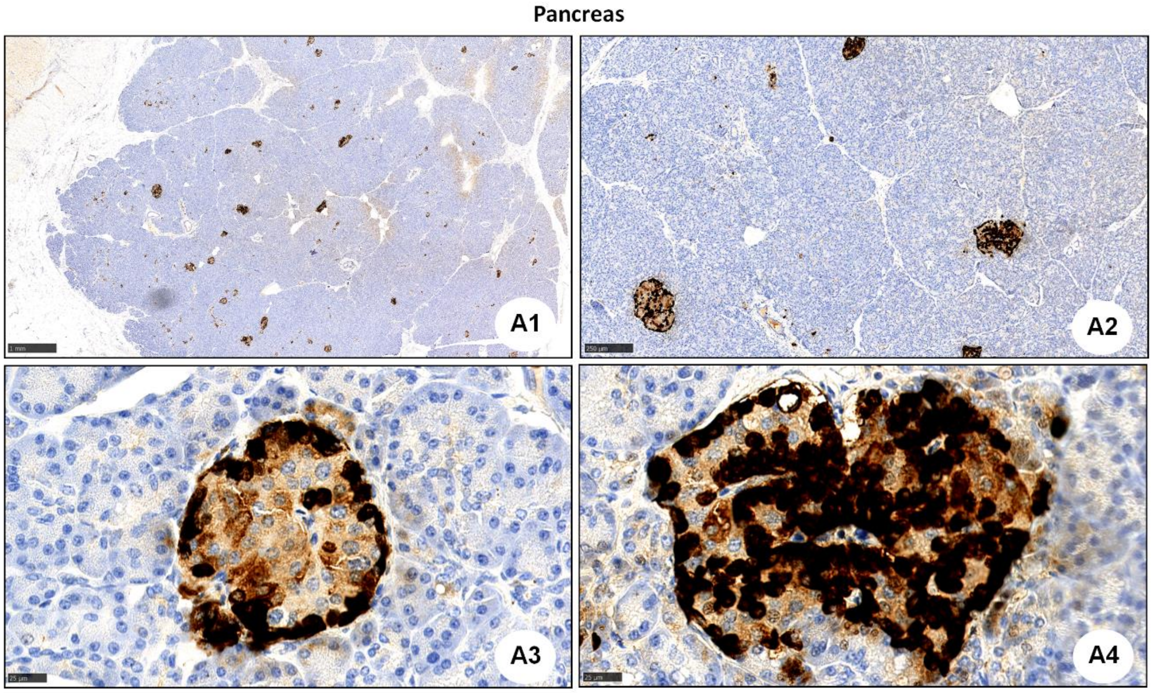

Suppl. Fig. 3. Immunohistochemical detection of OGR1. Localisation of OGR1 in healthy human pancreas, OGR1 strongly positive cells are confined to distinct focal points. Scale bars: **A1.** 1 mm; **A2.** 250 µm; detail images, **A3.** and **A4.** 25 µm.

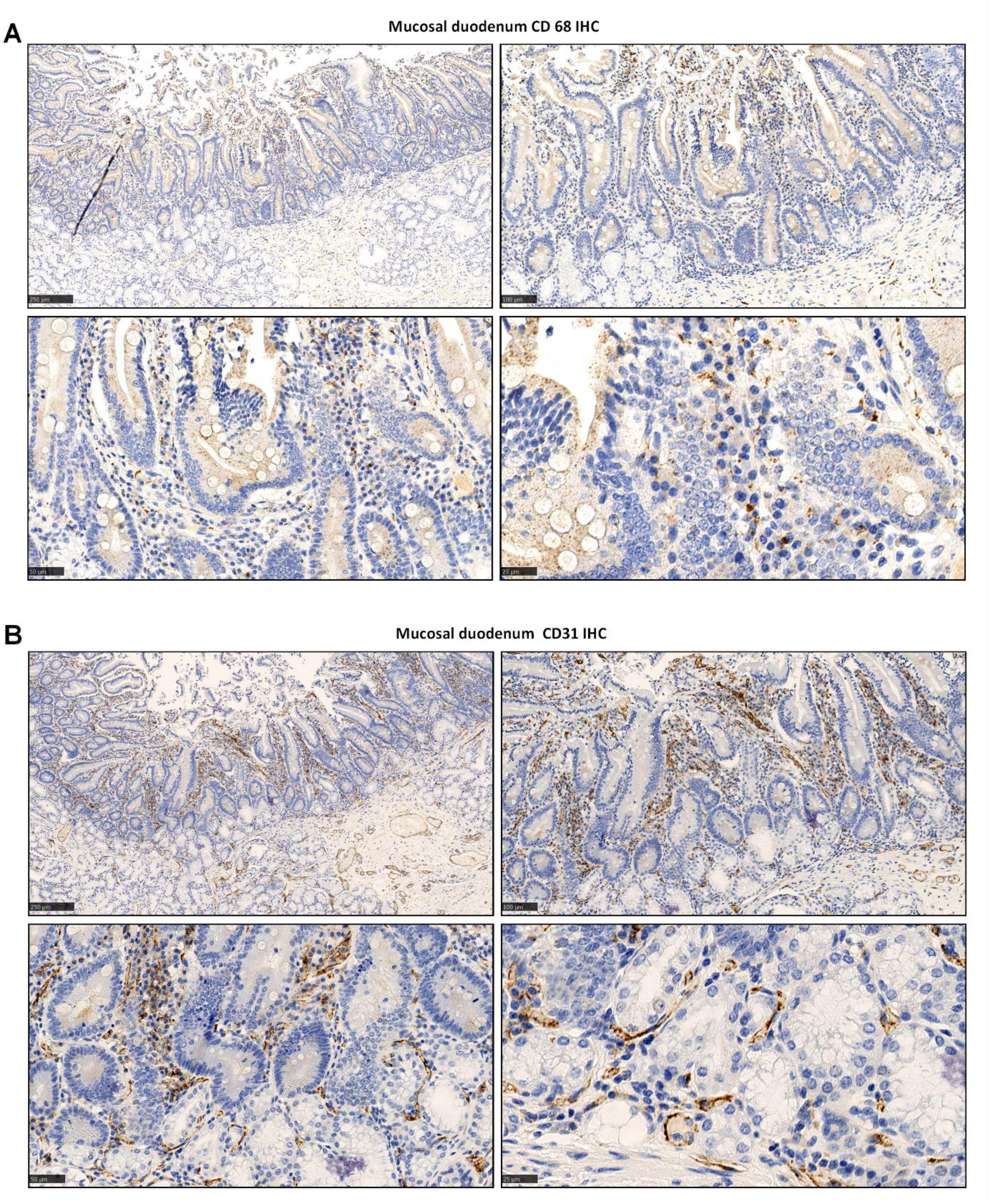

Suppl. Fig. 4. Immunohistochemical detection of CD68 and CD31 in healthy human mucosal duodenum. **A.** IHC staining (brown colour) with anti-CD68 antibody, counterstaining with haematoxylin. **B.** IHC staining (brown colour) with anti-CD31 antibody, counterstaining with haematoxylin. Scale bars: as indicated on images.

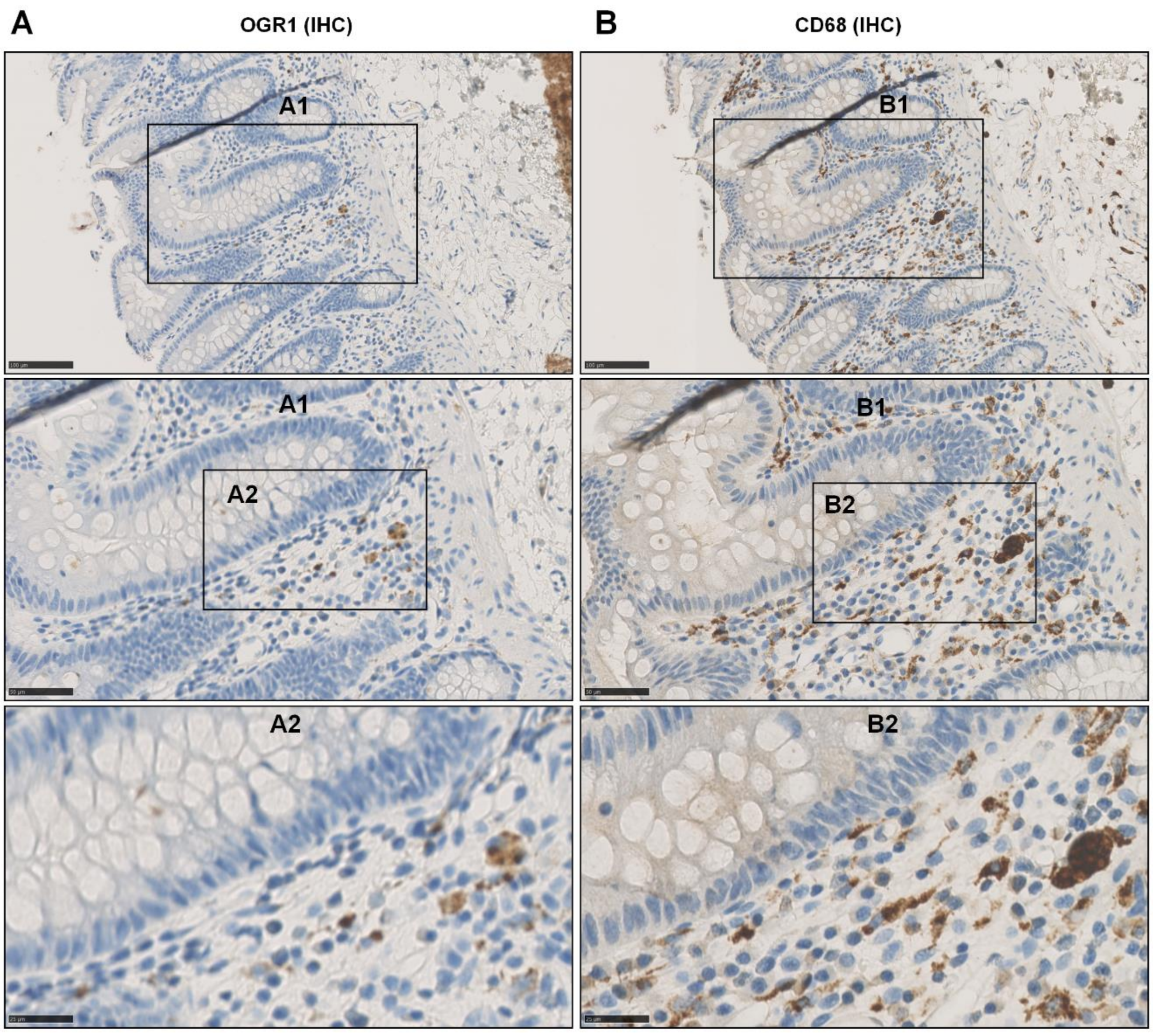

Suppl. Fig. 5. Immunohistochemical (IHC) detection of OGR1 and CD68 in colonic tissue. **A.** IHC staining (brown colour) with anti-OGR1 antibody, counterstaining with haematoxylin. **B.** IHC staining (brown colour) with anti-CD68 antibody, counterstaining with haematoxylin. Scale bars: as indicated on images. Intestinal samples were taken from 5 non-IBD subjects and paired (non-inflamed and inflamed) samples from 5 CD patients and 5 UC patients. Representative images are shown from a UC patient, non-inflamed tissue.

Suppl. Fig. 6

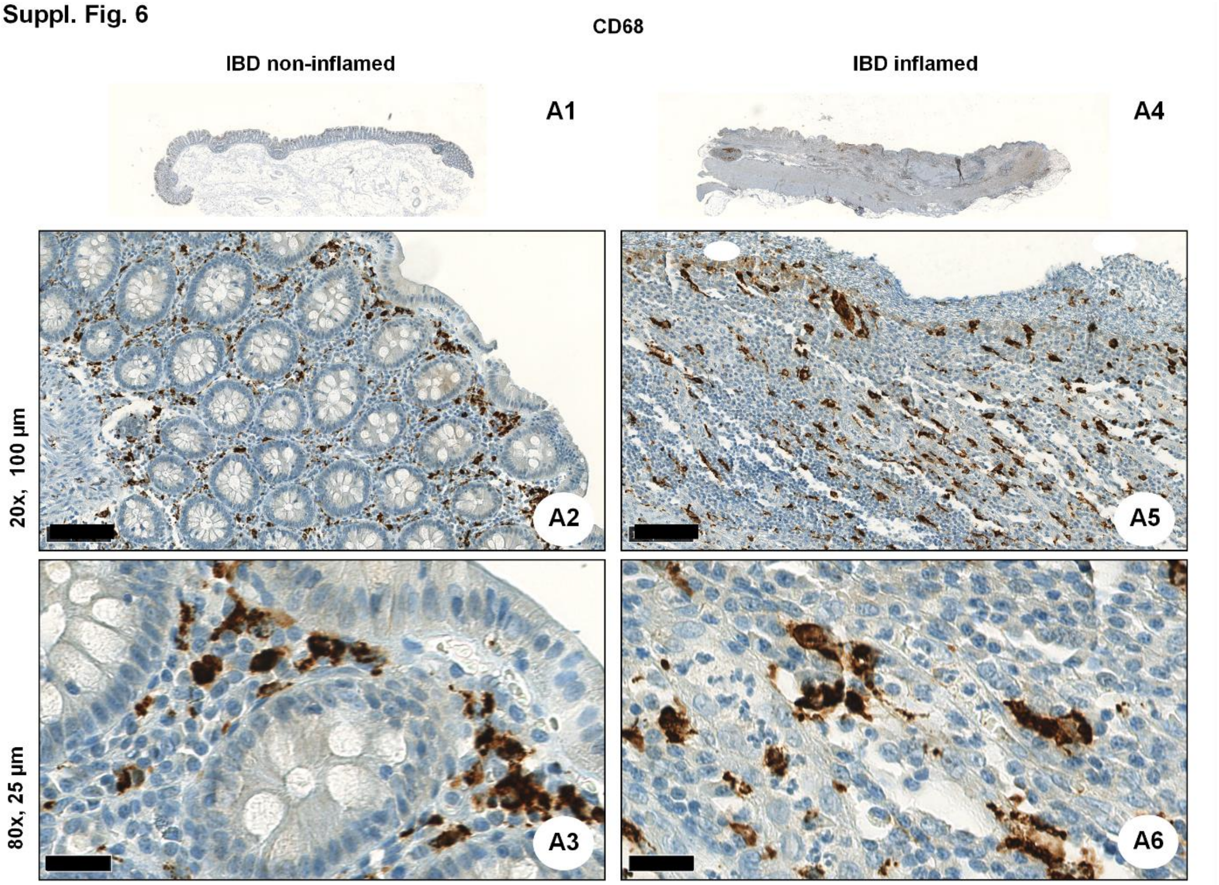

Suppl. Fig. 6. Immunohistochemical detection of CD68 and CD31 in non-inflamed and inflamed intestinal tissue. A. IHC staining (brown colour) with anti-CD68 antibody, counterstaining with haematoxylin. **A1–A3.** Non-inflamed, **A4–A6.** Inflamed. Intestinal samples were taken from 5 non-IBD subjects and paired (non-inflamed and inflamed) samples from 5 CD patients and 5 UC patients. Representative images shown are from a CD patient. Scale bars: **A2.** and **A5.** 100 μm; **A3**. and **A6.** 25 μm.

Suppl. Fig. 7

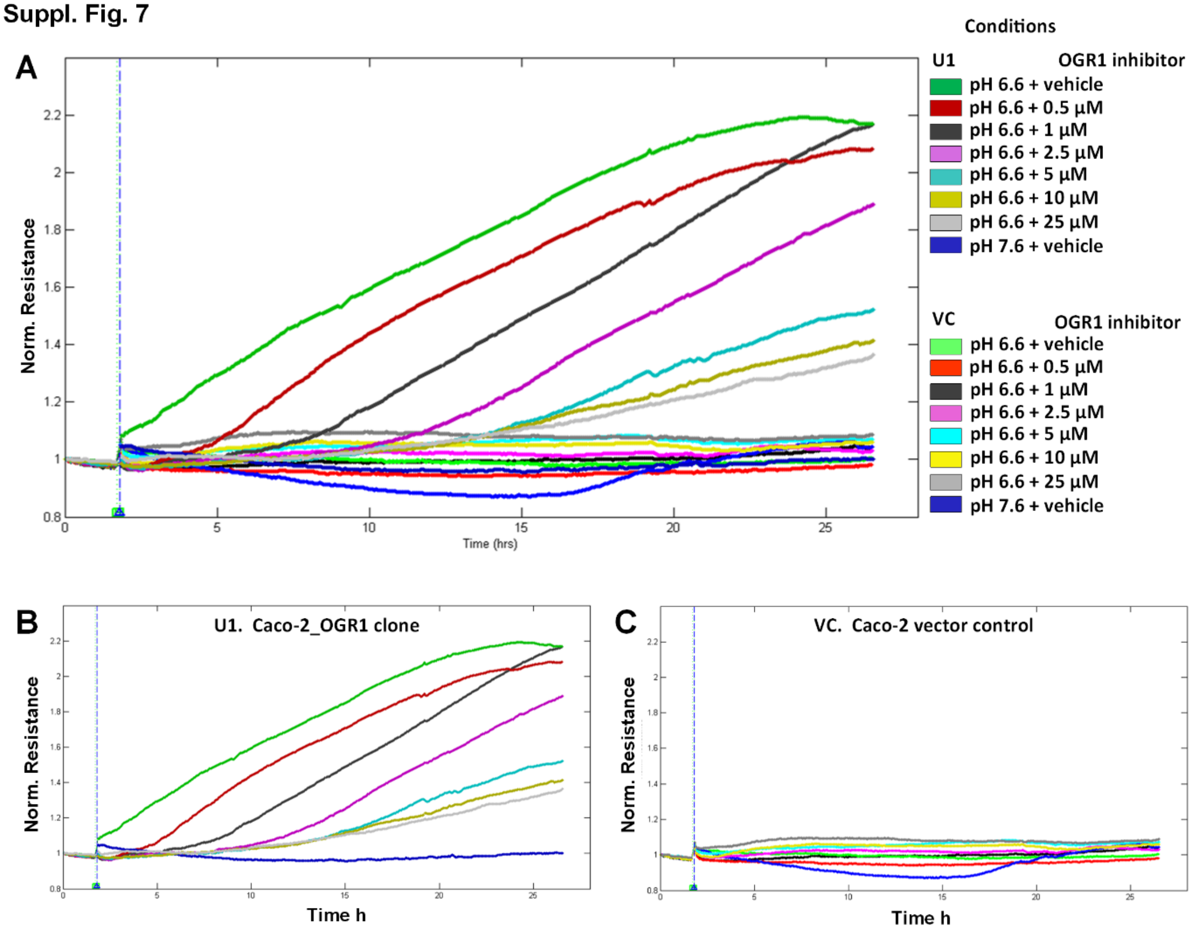

Suppl. Fig. 7. Efficacy of the OGR1 inhibitor was measured using electric cell-substrate impedance-sensing (ECIS) technology. Stably overexpressing OGR1 Caco-2 clone, (clone U1); negative control, Caco-2 vector control (VC) cells, grown to confluent monolayers, were subjected to an acidic pH shift with or without the OGR1 inhibitor (0.5, 1.0, 2.5, 5, 10, 25 µM). Changes in resistance of cell monolayers were monitored in real time. **A** Representative graph of 8 independent experiments is shown. **B.** Representative graph of one experiment with clone U1. **C.** Representative graph of one experiment with Caco-2 VC. OGR1: ovarian cancer G-protein-coupled receptor 1.
